# SRSF3, a Splicer of the *PKM* Gene, Regulates Cell Growth and Maintenance of Cancer-Specific Energy Metabolism in Colon Cancer Cells

**DOI:** 10.3390/ijms19103012

**Published:** 2018-10-02

**Authors:** Yuki Kuranaga, Nobuhiko Sugito, Haruka Shinohara, Takuya Tsujino, Kohei Taniguchi, Kazumasa Komura, Yuko Ito, Tomoyoshi Soga, Yukihiro Akao

**Affiliations:** 1 United Graduate School of Drug Discovery and Medical Information Sciences, Gifu University, 1-1 Yanagito, Gifu, Gifu 501-1193, Japan; v3501001@edu.gifu-u.ac.jp (Y.K.); v3501002@edu.gifu-u.ac.jp (N.S.); harukashinohara313@gmail.com (H.S.); 2 Department of Urology, Osaka Medical College, 2-7 Daigaku-machi, Takatsuki, Osaka 569-8686, Japan; uro061@osaka-med.ac.jp; 3 Department of General and Gastroenterological Surgery, Osaka Medical College, 2-7 Daigaku-machi, Takatsuki, Osaka 569-8686, Japan; 4 Translational Research Program, Osaka Medical College, 2-7 Daigaku-machi, Takatsuki, Osaka 569-8686, Japan; sur144@osaka-med.ac.jp (K.T.); uro051@osaka-med.ac.jp (K.K.); 5 Department of Anatomy and Cell Biology, Division of Life Sciences, Osaka Medical College, 2-7 Daigaku-machi, Takatsuki, Osaka 569-8686, Japan; an1006@osaka-med.ac.jp; 6 Institute for Advanced Biosciences, Keio University, 246-2 Mizukami, Kakuganji, Tsuruoka, Yamagata 997-0017, Japan; soga@sfc.keio.ac.jp

**Keywords:** energy metabolism, alternative splicing, oncogenes

## Abstract

Serine and arginine rich splicing factor 3 (SRSF3), an SR-rich family protein, has an oncogenic function in various kinds of cancer. However, the detailed mechanism of the function had not been previously clarified. Here, we showed that the SRSF3 splicer regulated the expression profile of the pyruvate kinase, which is one of the rate-limiting enzymes in glycolysis. Most cancer cells express pyruvate kinase muscle 2 (PKM2) dominantly to maintain a glycolysis-dominant energy metabolism. Overexpression of SRSF3, as well as that of another splicer, polypyrimidine tract binding protein 1 (PTBP1) and heterogeneous nuclear ribonucleoprotein A1 (hnRNPA1), in clinical cancer samples supported the notion that these proteins decreased the Pyruvate kinase muscle 1 (PKM1)/PKM2 ratio, which positively contributed to a glycolysis-dominant metabolism. The silencing of *SRSF3* in human colon cancer cells induced a marked growth inhibition in both in vitro and in vivo experiments and caused an increase in the PKM1/PKM2 ratio, thus resulting in a metabolic shift from glycolysis to oxidative phosphorylation. At the same time, the silenced cells were induced to undergo autophagy. SRSF3 contributed to PKM mRNA splicing by co-operating with PTBP1 and hnRNPA1, which was validated by the results of RNP immunoprecipitation (RIP) and immunoprecipitation (IP) experiments. These findings altogether indicated that SRSF3 as a *PKM* splicer played a positive role in cancer-specific energy metabolism.

## 1. Introduction

Most differentiated cells produce ATP efficiently by using both the tricarboxylic acid (TCA) cycle in mitochondria in the presence of oxygen and glycolysis under the condition of a relatively lower oxygen level. However, most cancer cells use glycolysis frequently even when enough oxygen is present [1]. This phenomenon, termed “the Warburg effect” or “aerobic glycolysis”, was first observed by Otto Heinrich Warburg in 1924 and is referred to as “the Warburg hypothesis” [2,3]. Pyruvate kinase (PK) is one of the important rate-limiting enzymes in glycolysis, catalyzing the conversion of phosphoenolpyruvate (PEP) and ADP to pyruvate and ATP [4]. In mammals, PK has four isoforms, that is, PKL, PKR, PKM1, and PKM2 [5,6,7]. PKL and PKR are encoded by the same single gene, *PKL*, and the expression of these isoforms is controlled by tissue-specific promoters [8]. PKL performs gluconeogenesis in tissues such as liver, kidney, and intestine, and its expression is regulated by nutrient conditions. PKR is expressed in erythrocytes and it is similar to PKL in terms of kinetic characteristics and regulation [9]. PKM1 is expressed in the tissues that require a large amount of ATP, such as brain and muscle, and it comprises four subunits having high affinity for PEP [10]. PKM2 is expressed in all proliferating cells, including tumor cells, and exists as two types of polymer: The tetrameric form with high affinity for PEP and the dimeric form having low affinity for it. PKM1 and PKM2 are encoded in the same single *PKM* gene: PKM1 lacks exon10 and PKM2, exon9, by alternative splicing (AS) to form their mature *PKM* mRNA [6]. The AS of primary mRNA is a molecular event that produces several mature-mRNA isoforms from a single primary mRNA [11]. AS is known to be a process that occurs in half of all human genes [12]. AS is regulated by several splicers, such as SR-rich family proteins and hnRNP family proteins; these are key factors of these splicers [13,14,15,16]. SRp20 (SRSF3), which is one of the most famous SR proteins and has been well studied, interacts with exonic splicing enhancer (ESE) sequences, thereby preventing exon skipping in pre-mRNA [11]. In particular, SRSF3 is known as one of the splicing factors of *PKM* gene, and it binds specifically to ESE on *PKM* exon 10 [17].

Recently, our group reported that the hnRNP family protein PTBP1, which is one of the splicers of *PKM*, is overexpressed in clinical tumor samples and is regulated by several onco-related genes, such as c-myc and klf-4, as well as by microRNA (miRs) -124, -143, -145, -1, and -133b in colorectal cancer [18,19,20], gastric cancer [21], and bladder cancer [22,23]. Moreover, PTBP1 promotes PKM2 expression in most cancers, thus maintaining the “Warburg effect” [4]. The decreased expression of PTBP1 induces partial switching of PKM isoform expression from PKM2 to PKM1, a phenomenon referred to as PKM switching [18,19,20,21,22,23]. However, the relationship between PKM switching and the splicer has not yet been determined. Moreover, it is unclear how each of them affects cancer-specific energy metabolism to promote cell proliferation and formation of malignant tumors. To clarify the role of the splicer in cancer metabolism, we examined how PKM isoform switching was induced by silencing each of the PKM splicer cording genes. In the current study, we focused on three proteins: PTBP1, hnRNPA1, already known as the PKM splicers [24], and SRSF3, one of the SR-rich family proteins. We showed that these splicer proteins coregulated PKM splicing, which was confirmed by IP and RIP experiments. Furthermore, siRNA for *SRSF3* (siR-*SRSF3*) exhibited a potent anti-proliferative activity. Consequently, silencing of *PTBP1*, *hnRNPA1*, and *SRSF3* resulted in increased levels of metabolites of the TCA cycle, as detected by metabolome analysis, after a partial metabolic shift from glycolysis to oxidative phosphorylation (OXPHOS). Our findings indicate that the PKM splicers of PTBP1, hnRNPA1, and SRSF3 were involved in the maintenance of cancer-specific metabolism and also tumorigenesis.

## 2. Results

### 2.1. Expression of PTBP1, hnRNPA1, and SRSF3 in Mouse Normal Tissues, Human Clinical Colorectal Tumors, and Human Cancer Cell Lines

**Figure 1:**
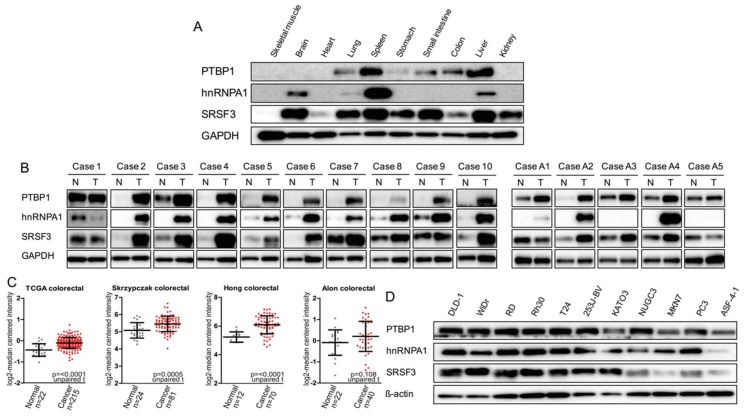
Expression profiles of polypyrimidine tract binding protein 1 (PTBP1), heterogeneous nuclear ribonucleoprotein A1 (hnRNPA1), and serine and arginine rich splicing factor 3 (SRSF3) in mouse normal tissues and colon tumor samples from the patients. (**A**) Western blot of PTBP1, hnRNPA1, and SRSF3 in normal mouse organs. PTBP1, hnRNPA1, SRSF3, and glyceraldehyde-3-phosphate dehydrogenase (GAPDH) were detected in the same membrane; (**B**) Western blot of three proteins in colon tumor samples from the patients. N: normal, T: tumor tissue. Cases 1–10 are cancer samples; and A1–A5, adenoma samples. PTBP1, hnRNPA1, SRSF3, and GAPDH were detected in the same membrane; (**C**) The SFRS3 mRNA expression level was examined for the indicated colorectal cancer cohorts. The unpaired T test was carried out to assess difference between the expression of SFRS3 mRNA in normal and cancer tissues. Error bar indicates standard deviation; (**D**) Western blot of these three proteins in the indicated cancer cell lines and in ASF-4-1 (human fibroblast) cells. PTBP1, hnRNPA1, SRSF3, and GAPDH were detected in the same membrane.

We firstly examined the expression profiles of the PTBP1, hnRNPA1, and SRSF3 in mouse normal tissues. Interestingly, PTBP1 was down-regulated in glucose-demanding organs, such as skeletal muscle, brain, and heart, and hnRNPA1 was expressed only in the brain, spleen, and liver. By contrast, SRSF3 was expressed in most organs/tissues, except skeletal muscle and heart. Thus, rather than hnRNPA1 and SRSF3, PTBP1 closely associated with energy metabolism, because PTBP1 was down-regulated extremely in brain and muscle tissues (Figure 1A). Next, we examined protein expression levels of PTBP1, hnRNPA1, and SRSF3 in clinical colorectal tumor samples. These three proteins were overexpressed in the tumor samples compared to those of the adjacent normal samples taken from the same colorectal cancer and adenoma cases (Figure 1B). These findings suggested that these three proteins may play a positive role in colorectal tumor development. To further assess the clinical relevance of these results, we analyzed publicly available gene expression profile data from the Oncomine database. As shown in Figure 1C, the *SRSF3* mRNA expression was significantly increased in colorectal tumor samples [25,26,27,28]. On the other hand, in all cancer cell lines tested and in human fibroblast ASF-4-1 cell line, PTBP1 was fairly expressed, and good expression of hnRNPA1 and SRSF3 was observed in most of the cancer cell lines (Figure 1D). In the ASF-4-1 cell line as a normal cell, the expression levels of PTBP1, hnRNPA1, and SRSF3 were lower than those of all cancer cell lines tested.

### 2.2. PKM Isoform Expression and Contribution of PTBP1, hnRNPA1, and SRSF3 to Tumorigenesis

**Figure 2:**
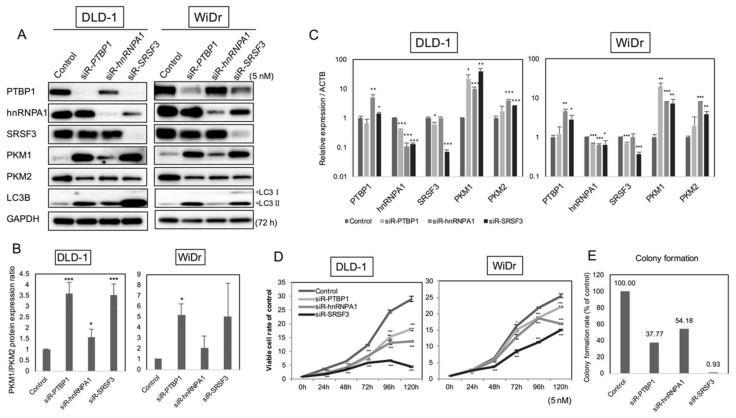
Effects on *PKM* gene and protein expression by silencing of *PTBP1*, *hnRNPA1*, or *SRSF3* in colorectal cancer cell lines DLD-1 and WiDr. (**A**) Western blot of PTBP1, hnRNPA1, SRSF3, PKM proteins, and LC3B prepared at 72 h after transfection of DLD-1 and WiDr cells with short-interfering RNA (siRNA) for *PTBP1*, *hnRNPA1*, and *SRSF3* (5 nM). PTBP1, hnRNPA1, SRSF3, and GAPDH were detected in the same membrane; (**B**) PKM1/PKM2 ratio calculated by densitometric values of PKM1 and PKM2 in (A). Control is indicated as 1.0; (**C**) mRNA expression levels in DLD-1 and WiDr cells at 72 h after transfection with each siRNA (5 nM); (**D**) Time-dependent cell growth of DLD-1 and WiDr cells transfected with each siRNA (5 nM), as determined by performing trypan blue staining; (**E**) Results of colony formation assay for DLD-1 cells transfected with each siRNA (5 nM). * *p* < 0.05, ** *p* < 0.01, *** *p* < 0.001 (Student’s *t* test).

In order to clarify the relationship between the expression profiles of three proteins and *PKM* mRNA splicing, we examined the expression levels of these three proteins and PKMs by silencing each protein cording gene in colon cancer DLD-1 and WiDr cells. As shown in Figure 2A,B, the silencing of *PTBP1*, *hnRNPA1*, and *SRSF3* consistently increased the ratio of PKM1/PKM2 in both DLD-1 and WiDr cells. At the same time, the transition of LC3B-I to LC3B-II as an indication of autophagy was observed by Western blot analysis (Figure 2A). Additionally, the relative mRNA expression level of *PKM1* almost paralleled the profiles of protein expression as shown in Figure 2A (Figure 2C).

In order to determine whether PTBP1, hnRNPA1, and SRSF3 have an oncogenic function, we examined cell-growth inhibition in vitro and soft-agar colony formation after silencing each gene. As a result, silencing of each gene dramatically inhibited the growth of DLD-1 and WiDr cells (Figure 2D). Among the siRNAs for each gene, siR-*SRSF3* especially showed the highest anti-proliferative activity. Furthermore, in the colony formation assay, siR-*SRSF3* also markedly decreased the soft-agar colony number compared to the others (Figure 2E).

**Figure 3:**
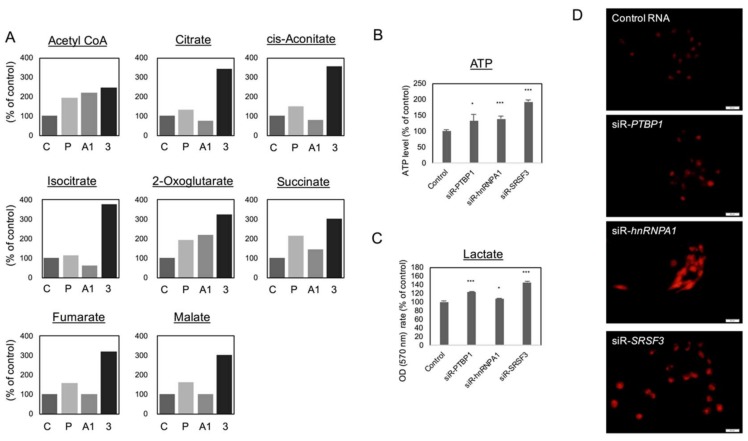
Effects on DLD-1 cellular metabolism of silencing of *PTBP1*, *hnRNPA1*, or *SRSF3*. (**A**) Metabolome analysis of DLD-1 cells transfected with siRNA for *PTBP1*, *hnRNPA1*, and *SRSF3* (5 nM) for 72 h (C, Control RNA; P, siR-*PTBP1*; A1, siR-*hnRNPA1*; 3, siR-*SRSF3*); (**B**) ATP assay for DLD-1 cells transfected with each siRNA (5 nM) for 72 h; (**C**) Lactate assay for DLD-1 cells transfected with each siRNA (5 nM) for 72 h; (**D**) Reactive Oxygen Species (ROS) assay using Mitosox. DLD-1 cells were transfected with each siRNA (5 nM) for 72 h. Scale bar; 20 µm. * *p* < 0.05; *** *p* < 0.001 (Student’s *t* test).

Since silencing of *PTBP1*, *hnRNPA1*, or *SRSF3* up-regulated the PKM1/PKM2 ratios, we performed metabolome analysis to ascertain the metabolic shift from glycolysis to OXPHOS. The metabolome analysis of DLD-1 cells indicated that the levels of metabolites in the TCA cycles were generally increased after the shift of PKM1/PKM2 ratios from PKM2 dominant to PKM1 dominant, which revealed the process of OXPHOS after the silencing of *PTBP1*, *hnRNPA1*, and *SRSF3* (Figure 3A). Furthermore, the levels of both intracellular ATP and lactate were increased in these cells (Figure 3B,C). As for Reactive Oxygen Species (ROS) production elicited by the increased OXPHOS, we were able to detect a significant increase in the ROS level, which was estimated by performing MitoSOX staining (Figure 3D). These results indicated that PTBP1, hnRNPA1, and SRSF3 were required for the maintenance of aerobic glycolysis by dominant expression of PKM2 rather than PKM1.

### 2.3. Silencing PTBP1, hnRNPA1, and SRSF3 Caused ROS-Induced Autophagic Cell Death in Colon Cancer Cells

**Figure 4:**
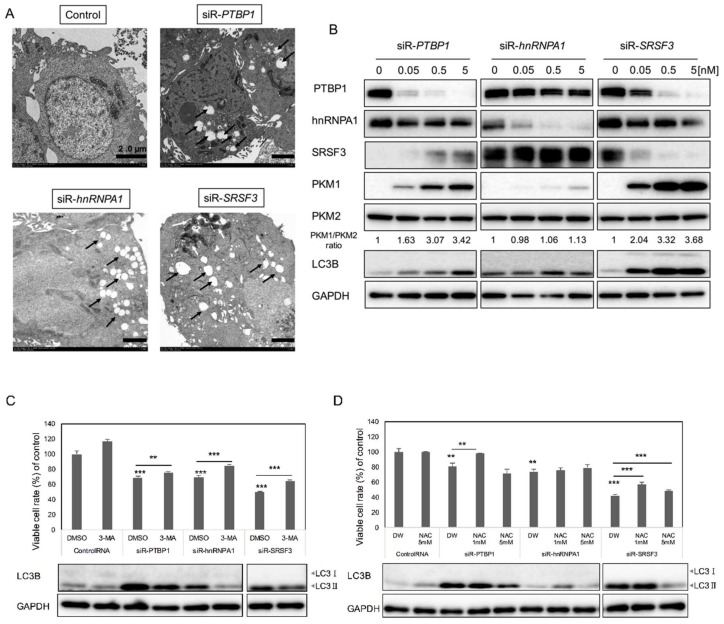
Silencing of *PTBP1*, *hnRNPA1*, or *SRSF3* induced autophagy in DLD-1 cells. (**A**) Morphological study by electron microscopy. DLD-1 cells were transfected with siRNA for *PTBP1*, *hnRNPA1*, and *SRSF3* (5 nM) for 72 h. Scale bar: 2.0 µm, black arrow: Autophagosome, white arrow: Mitophagy; (**B**) Concentration-dependence expression profiles of PTBP1, hnRNPA1, SRSF3, PKM isoforms, and LC3B at 72 h after transfection of DLD-1 cells with siRNA for *PTBP1*, *hnRNPA1*, and *SRSF3*, estimated by Western blot. PTBP1, hnRNPA1, SRSF3, LC3B and GAPDH, PKM1 and PKM2 were detected in the same membrane, respectively; (**C**) Cell viability and Western blot of LC3B after combination treatment with autophagy inhibitor 3-MA and each siRNA. DLD-1 cells were pre-treated with 3-MA (0.2 mM) for 5 h before transfection with each siRNA (5 nM) for 72 h; (**D**) Cell viability and Western blot of LC3B after combination treatment with autophagy inhibitor NAC and each siRNA. DLD-1 cells were treated with NAC (1, 5 µM) at 24 h after the start of transfection with each siRNA for 72 h. ** *p* < 0.01; *** *p* < 0.001 (Student’s *t* test).

With regards to the autophagy shown by biochemical analysis (Figure 2A, LC3B-I to LC3B-II), we also examined the morphological characteristics of autophagy promoted by each siRNA using electron microscopy. As shown in Figure 4A, the typical findings, such as autophagosome and autolysosome formation, were observed in the cells treated with the siRNA (Figure 4A, black arrows). Especially in the cells treated with siRNA for *SRSF3*, mitophagy was evident (Figure 4A, white arrows in siR-*SRSF3* panel). The autophagy was induced by silencing of *PTBP1*, *hnRNPA1*, or *SRSF3* in a concentration-dependent manner. Moreover, the ratios of PKM1/PKM2 estimated by Western blot analysis were also increased in a concentration-dependent manner (Figure 4B). The autophagy inhibitor 3-MA prevented the decrease in viable cell numbers elicited by treatment with siRNAs for the *PTBP1*, *hnRNPA1*, or *SRSF3*, which was also confirmed by Western blotting of LC3B (Figure 4C). In order to validate the increased ROS production after the treatment with each siRNA for each gene, we examined the cell growth in the presence of an ROS inhibitor, N-acetyl cysteine (NAC). NAC partially reversed the decrease in viable cell numbers elicited by siRNAs for each gene, and the LC3B transition from I to II was also reversed by the treatment with NAC (Figure 4D). These results altogether suggested that the increase in the PKM1/PKM2 expression ratio by silencing each gene caused the autophagic cell death, along with increased levels of ROS.

### 2.4. Anti-Tumor Effect by Silencing SRSF3 in Vivo

**Figure 5:**
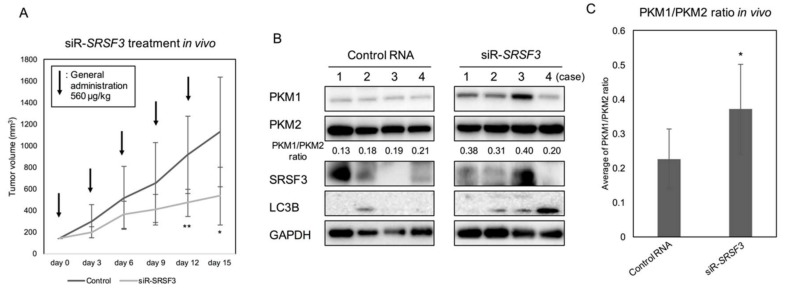
Effect on tumor of silencing of SRSF3 in colorectal cancer cell line DLD-1 xenograft model mice in vivo. (**A**) Time-course of tumor growth in mice intravenously injected with control RNA or siR-SRSF3 (560 µg/kg) carried by nanopolymer. Arrow, general injection; (**B**) Western blot of PKM isoforms, SRSF3, and LC3B in xenograft tumor samples from control RNA- or siR-*SRSF3* treated mice. Numbers represent the ratios of PKM1/PKM2 calculated by densitometric values of PKM1 and PKM2. The representative 4 cases were shown; (**C**) Average values of PKM1/PKM2 ratio in control and siR-*SRSF3* treated groups were indicated (B) (*n* = 8). * *p* < 0.05; ** *p* < 0.01 (Student’s *t* test).

In order to further validate the anti-tumor effect of siR-*SRSF3* in vivo, we administered siR-*SRSF3* to DLD-1 cell-xenografted nude mice. After subcutaneous implantation of DLD-1 cells into the back of the mice, we sorted the mice into 2 different groups, control siRNA and siR-*SRSF3*, after the xenografted tumors had reached a volume of 100 mm^3^. After general administration with the siRNA every 72 h (totally 2.8 mg/kg), the tumor growth was significantly suppressed in the siR-*SRSF3*-treated group compared to that in the control one (Figure 5A). The PKM1/PKM2 ratios in the samples from xenografted tumors significantly increased in the siR-*SRSF3* treated group compared to those in the control one, as estimated by Western blot analysis (Figure 5B,C). These in vivo data indicated that *SRSF3* could have an oncogenic role, as evaluated in in vitro experiments.

### 2.5. PTBP1 and SRSF3 Interacted with PKM mRNA

**Figure 6:**
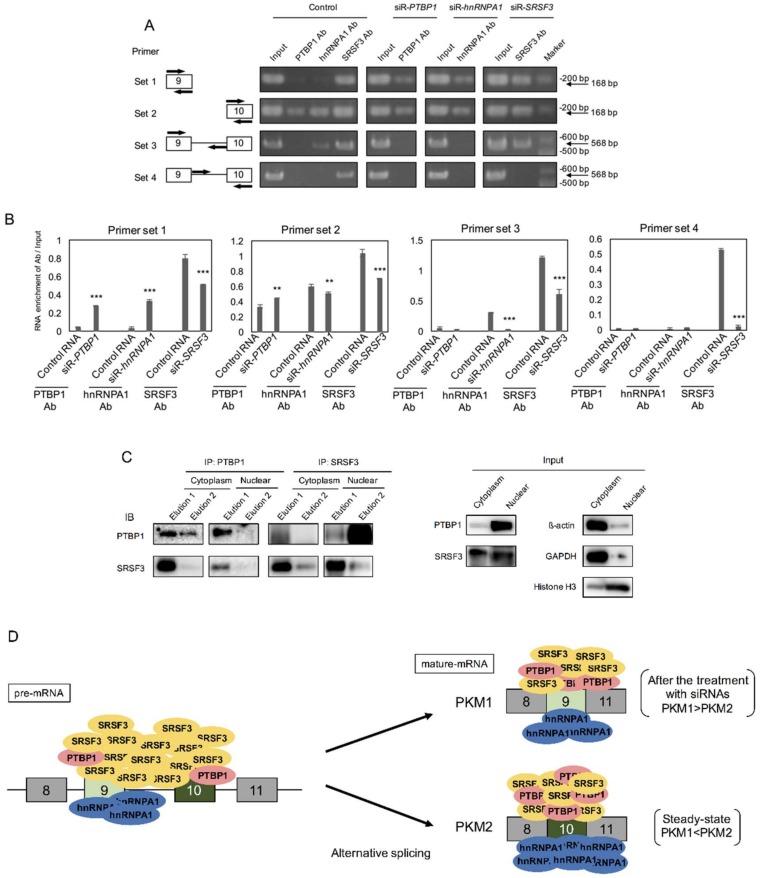
Molecular relationship among SRSF3, PTBP1, and *PKM* mRNA. (**A**)Amounts of PCR-fragments from *PKM1* and *PKM2* mRNAs obtained by RIP assay. The samples are from the cells treated with each siRNA. The detail is shown in the Materials and Methods section; (**B**) Densitometric values of the bands of (A); (**C**) Western blot of PTBP1 and SRSF3 proteins from immunoprecipitation assay samples; (**D**) Scheme of relationship between splicing factors, PTBP1, hnRNPA1, and SRSF3, and *PKM* mRNA. ** *p* < 0.01; *** *p* < 0.001 (Student’s *t* test).

Next, in order to validate the molecular relationship between the PKM splicer of PTBP1, hnRNPA1, and SRSF3 and *PKM* mRNA, we performed RNP immunoprecipitation (RIP) assay by using an antibody against each protein. PTBP1 has already been ascertained to be a splicer of the *PKM* gene [29]. The results of RIP assay indicated that hnRNPA1 and SRSF3 interacted with *PKM* pre-mRNA and mature-mRNA for *PKM* alternative splicing. PTBP1 mainly interacted with *PKM* mature-mRNA (Figure 6A,B). As for the down-regulation of PTBP1 after siR-*SRSF3* introduction (Figure 2A), we performed immunoprecipitation (IP) assays using DLD-1 cells to clarify protein–protein interaction between PTBP1 and SRSF3. As a result, we found that PTBP1 and SRSF3 interacted with each other in both cytoplasm and nuclear fractions (Figure 6C). As for hnRNPA1, we could detect the interaction between hnRNPA1 and PTBP1 or SRSF3 only in a cytoplasmic fraction, but not in a nuclear fraction (Figure S1). These results indicated that PTBP1 and SRSF3 were members of the PKM splicer complex that executed the splicing of the *PKM* gene (Figure 6D).

## 3. Discussion

In the current study, we showed that gene silencing of *PTBP1*, *hnRNPA1*, and *SRSF3*, which for coding proteins is known as the splicing factor, induced a partial PKM switching from PKM2 dominant to PKM1 dominant, followed by a metabolic shift from glycolysis to OXPHOS in the TCA cycle in the cancer cells tested. Finally, the cancer cells produced a certain amount of ROS, which resulted in the induction of mitophagy and/or autophagic cell death. We could validate a relationship between splicer proteins and *PKM* by RIP assay and also validate it between PTBP1 and SRSF3 by IP assays. The relationship between these proteins was also considered. As shown in Figure 6D, PTBP1, hnRNPA1, and SRSF3 related to the splicing event of *PKM*. PTBP1 and hnRNPA1 bound to exon 10 in steady state. HnRNPA1 bound to sites from exon 9 to just before exon 10. However, PTBP1 and hnRNPA1 also bound to exons 9 and 10 after treatment with each siRNA. These results suggested that PTBP1 and hnRNPA1 played almost similar roles in the splicing. On the other hand, SRSF3 bound to anywhere between exons 9 and 10. After treatment with siR-*SRSF3*, SRSF3 did not operate the site just after exon 9 to exon 10. SR-rich protein interacted with ESE sequences, thereby preventing exon skipping in pre-mRNA [11]. These results indicated that SRSF3 prevented exon 10 skipping in steady state of cancer cells. Therefore, it is suggested that these splicers may act depending on the stage in development of tumor.

Our findings indicated a positive contribution of splicing factor of PTBP1, hnRNPA1, and SRSF3 to PKM2 dominant expression by AS, resulting in maintenance of the “Warburg effect”. Among PTBP1, hnRNPA1, and SRSF3 examined, SRSF3 exhibited a potent oncogenic function that was demonstrated by anchorage-independent growth. These three proteins were overexpressed in certain clinical colorectal tumor and adenoma samples, and also in the several cancer cell lines tested. Consequently, these three proteins contributed to the promotion of cell growth through the regulation of energy metabolism to use glycolysis efficiently. In particular, the cell silenced *SRSF3* by intravenously injected siR-*SRSF3* inhibited tumor growth in DLD-1 cell-xenografted mice.

Interestingly, the silencing of *SRSF3* decreased the protein expression levels of PTBP1 and hnRNPA1 in both DLD-1 and WiDr cells. It has been reported that SRSF3 regulates the expression of PTBP1 and PTBP2 in HEK-293 (human embryonic kidney epithelial cell) and CAL 27 (oral squamous cell carcinoma) cells [30]. It is also speculated that SRSF3 may be associated with epigenetic change, such as post-translational modification by microRNA (Figure 2C). Additionally, the silencing of *SRSF3* induced autophagic cell death. It is suggested that the decrease expression of SRSF3 induced cell senescence through p53ß mRNA and protein expression [31].

It was previously reported by Rong Jia et al. [32] and by Kim, Park, and Jeong [15,33,34] that SRSF3 has an oncogenic role in carcinogenesis of various kinds of cancer. SRSF3 regulates the expression of Forkhead box protein M1 (FOXM1) and two of its transcriptional targets, PLK1 and Cdc25B, which control cell-cycle progression from the G2 to M phase [32]. SRSF3 represses the expression of programmed cell death 4 (PDCD4) protein, which is a tumor suppressor involved in apoptosis, and PDCD4 down-regulation is a potential marker for solid tumor prognoses [15,33,34]. These studies supported our observations that SRSF3 had an oncogenic role.

Chen et al. reported that high expression levels of hnRNPA1/A2 and PTBP1, which are an intronic splicing silencer (ISS), were bound to intron 9, leading to exon 9 exclusion in *PKM* mRNA in cancer [35]. However, low expression levels of these proteins lead to binding to intronic sites, resulting in exon 9 inclusion and exon 10 exclusion, and these splicer proteins have multiple functions, according to these expression levels [24,35]. Additionally, Zhenxun Wang et al. reported that SRSF3 binds to a splicing enhancer element in the exon 10 of *PKM* mRNA, which promotes to process *PKM2* mRNAs [17]. Our findings support part of the evidence described by their reports. Moreover, SRSF3 may act as multiple players, such as an exonic splicing silencer (ESS), ESE, ISS, and intronic splicing enhancer (ISE) (Figure 6A,B).

Furthermore, cDNA microarray data indicated that SRSF3 extremely up-regulated *RAS* expression. *KRAS* is proto-oncogene that has a substantial role in many growth-related signaling pathways. Approximately one third of colorectal cancer patients have a point mutation in their *KRAS* gene, which causes constitutional activation of the RAS signaling networks [36]. In future work, we will clarify the relationship between SRSF3 and RAS expression networks.

In the current study, we demonstrated that the hnRNP family members PTBP1 and hnRNPA1, as well as the SR-rich protein family member SRSF3, regulated the PKM2-dominant expression, and that the silencing of *SRSF3* induced a marked growth inhibition through the metabolic shift in part from glycolysis to OXPHOS by increasing the PKM1/PKM2 ratio. Our results strongly suggest that PTBP1, hnRNPA1, and SRSF3 controlled cancer-specific energy metabolism at least in part through affecting the *PKM* gene expression profile via AS, which was clarified by RIP and IP assays, and that these proteins coding RNA would be targets for RNA medicine.

## 4. Materials and Methods

### 4.1. Patients and Samples

All human samples were obtained from patients who had undergone biopsy or surgery for resection at Fujita Health University Hospital (Toyoake, Aichi, Japan), Gifu University Hospital (Gifu, Gifu, Japan) or Misao Health Clinics (Gifu, Gifu, Japan). Collection and distribution of the samples was approved by each of the appropriate institutional review boards in accordance with the Declaration of Helsinki. (Approval number: 09-152 (25 November 2009), Fujita Health University; 28-508 (23 March 2017), Gifu University) Of the paired adenoma samples, 1 was obtained from Misao Health Clinics and 4 from Fujita Health University Hospital. All patients with previously untreated (or recently diagnosed) colorectal cancer were selected. The characteristics of the patients are shown in Table 1 and Table 2. Under a pathologist’s supervision, all tissue sample pairs were collected from surgically resected tissues, with these paired samples being from the primary tumor and its adjacent non-tumor mucosal tissue in the same patient.

### 4.2. Dataset Analysis

The mRNA expression levels in clinically available datasets were examined using Oncomine (https://www.oncomine.org/resource/login.html).

### 4.3. Cell Culture and Cell Viability

Human colorectal cancer cell lines DLD-1 and WiDr, human rhabdmyosarcoma cell line Rh30, human urinary bladder carcinoma cell lines T24 and 253J-BV, human stomach cancer cell lines KATOIII, NUGC3, and MKN7, and human prostate cancer cell line PC3 were maintained in RPMI-1640 medium (Wako Pure Chemical Industries, Ltd., Osaka, Japan), supplemented with 8% (*v*/*v*) heat-inactivated FBS (Sigma-Aldrich Co., St. Louis, MO, USA) and 2 mM l-glutamine. Human diploid fibroblast cell line ASF-4-1 and human rhabdmyosarcoma cell line RD were maintained in E-MEM medium (Wako), supplemented with 10% (*v*/*v*) heat-inactivated FBS and 2 mM l-glutamine. All cell lines were cultured under an atmosphere of 95% air and 5% CO_2_ at 37 °C. DLD-1, WiDr, T24, 253J-BV, KATOIII, MKN7, RD, and ASF-4-1 cell lines were obtained from the JCRB cell bank (Osaka, Japan). The PC3 cell line was purchased from the American Type Cell Collection (ATCC). The Rh30 and NUGC3 cell lines were provided by Dr. Hosoi (Kyoto Prefectural University of Medicine) and Taiho Pharmaceutical (Tokyo, Japan), respectively. The number of viable cells was determined by performing the trypan-blue (Life Technologies, Carlsbad, CA, USA) dye exclusion test.

### 4.4. Transfection with Short-Interfering RNA for Each Splicer Cording Gene

DLD-1 or WiDr cells were seeded at a concentration of 0.5 × 10^5^ cells/ml on the day before transfection. Short-interfering RNA (siRNA; siR-) for *PTBP1* and *SRSF3* were designed and synthesized by Life Technologies (Carlsbad, CA, USA), and *hnRNPA1* was purchased from Santa Cruz Biotechnology (Santa Cruz, CA, USA), which were used for the transfection of the cells, which was achieved using cationic liposomes, Lipofectamine RNAiMAX (Life Technologies), according to the manufacturer’s protocol. The nonspecific control siRNA (HSS, Hokkaido, Japan) sequence was 5′-GUAGGAGUAGUGAAAGGCC-3′. The sequences of siR-*PTBP1* and -*SRSF3* were 5′-AUCUCUGGUCUGCUAAGGUCACUUC-3′ and 5′-AGAACACUAUGUGGCUGCCGUGUAA-3′, respectively. The effects which manifested through the introduction of siRNAs into the cells were assessed at 72 h after the transfection.

### 4.5. Inhibitor Agents

We used lysosome inhibitor (3-MA) and free-radical scavenger *N*-acetyl-l-cysteine (NAC), which were purchased from EMD Chemicals, Inc. (San Diego, CA, USA) and Sigma Aldrich, respectively. We added 3-MA (0.2 mM) to the culture medium 5 h before the transfection with siRNA for each splicer cording genes. NAC (1 mM, 5 mM) was added to the culture medium 24 h before the transfection with control RNA, siR-*PTBP1*, siR-*hnRNPA1*, or siR-*SRSF3*.

### 4.6. Protein Extraction and Western Blot Analysis

Protein extraction and Western blot experiments were performed as described previously [37]. Primary antibodies against the following antigens were used: PTBP1, hnRNPA1, and LC3B (Cell Signaling Technology, Danvers, MA, USA); PKM1 and PKM2 (Novus Biologicals, Littleton, CO, USA); SRSF3 (SRp20) (MBL, Nagoya, Japan). The 2nd antibodies used were HRP-conjugated horse anti-mouse or goat anti-rabbit IgG antibody (Cell Signaling Technology). The quantity loaded was verified by re-incubating the same membrane with anti-β-actin antibody (Sigma-Aldrich) or anti-GAPDH (Cell Signaling Technology).

### 4.7. RNA Extraction and Real-Time Reverse Transcription-PCR

Total RNA was isolated from cultured cells using a NucleoSpin miRNA isolation kit (MACHEREY-NAGEL, GmbH & Co. KG, Düren, Germany). For determination of the expression of the expression levels of *PTBP1*, *hnRNPA1*, *SRSF3*, *PKM1*, *PKM2,* and *GAPDH* mRNAs, total RNA was reverse-transcribed with a PrimeScript RT Reagent Kit (Takara Bio Inc., Otsu, Japan). Real-time PCR was then performed with specific primers and THUNDERBIRD SYBR qPCR Mix (TOYOBO, Osaka, Japan). The primers for PTBP1, hnRNPA1, SRSF3, PKM1, PKM2, and GAPDH were the following: *PTBP1*-Forward, 5′-ATCAGGCCTTCATCGAGATGCACA-3′; and *PTBP1*-Reverse, 5′-TGTCTTGAGCTCCTTGTGGTTGGA-3′; *hnRNPA1*-Forward, 5′-ACATATGCCACTGTGGAGGAG-3′; and *hnRNPA1*-Reverse, 5′-CTTGCTTTGACAGGGCTTTTC-3′; *SRSF3*-Forward, 5′-GGCAATCTTGGAAACAATGG-3′; and *SRSF3*-Reverse, 5′-TTCACCATTCGACAGTTCCA-3′; *PKM1*-Forward, 5′-TGAAGAACTTGTGCGAGCCT-3′; and *PKM1*-Reverse, 5′-GCCAGACTCCGTCAGAACTA-3′; *PKM2*-Forward, 5′-TTACCAGCGACCCCACAGAA-3′; and *PKM2*-Reverse, 5′-GACGATTATGGCCCCACTGC-3′; *GAPDH*-Forward, 5′-CCACCCATGGCAAATTCCATGGCA-3′; and *GAPDH*-Reverse, 5′-TCTAGACGGCAGGTCAGGTCCACC-3′. The relative expression levels were calculated using the ∆∆*C*_t_ method. *GAPDH* was used as an endogenous normalizer for *PTBP1*, *hnRNPA1*, *SRSF3*, *PKM1*, and *PKM2* expression levels.

### 4.8. Soft Agar Colony Formation Assay

DLD-1 cells were seeded at a concentration of 0.5 × 10^5^ cells/ml in 6-well plates. Twenty-four hours later, the cells were transfected with control RNA, siR-*PTBP1*, siR-*hnRNPA1*, or siR-*SRSF3* (5 nM) for 48 h. Next, 2 × 10^2^ cells from each transfection group were suspended in 0.4% agarose (Lonza, ME, USA) in RPMI 1680 medium (Wako), supplemented with 8% (*v*/*v*) heat-inactivated FBS (Sigma-Aldrich) in 6-cm dishes (CORNING, Corning, NY, USA) as the upper layer, with 0.5% agarose in RPMI 1680 medium, supplemented with 8% (*v*/*v*) heat-inactivated FBS as the bottom layer. The cells were cultured under an atmosphere of 95% air and 5% CO_2_ at 37 °C for 2 weeks. The colonies were fixed with methanol (Sigma Aldrich) for 10 min and stained with 5% Giemsa’s stain solution (MUTO PURE CHEMICALS CO., LTD., Tokyo, Japan) for 30 min. After wash and dry, the colonies were counted.

### 4.9. Metabolome Analysis

DLD-1 cells were seeded at a concentration of 0.5 × 10^5^ cells/ml in 6-well plates on the day before the transfection. After 24 h, the cells were transfected with 5 nM control RNA, siR-*PTBP1*, siR-*hnRNPA1*, or siR-*SRSF3* for 72 h under an atmosphere of 95% air and 5% CO_2_ at 37 °C. The procedure for sample preparation for metabolome analysis was previously described [38,39]. Briefly, after the cells had been washed twice with ice-cold 5% mannitol, metabolites were extracted with 1 mL of ice-cold methanol containing internal standards: A 25 mM concentration of each of methionine sulfone (Met-Sul; Wako, Osaka, Japan), 2-(*N*-morpholino)ethanesulfonic acid (MES; Wako), and D-camphor-10-sulfonic acid (CSA; Wako). Four-hundred microliters of collected extracts were transferred into another tube, mixed with 400 mL of chloroform and 200 mL of Milli-Q water, and centrifuged at 10,000 *g* for 3 min at 4 °C. A 400-mL aliquot of the aqueous layer was centrifugally filtered through a 5-kDa cut-off membrane (UltrafreeMC-PLHCC for Metabolome Analysis; Human Metabolome Technologies, Tsuruoka, Yamagata, Japan) to remove proteins from the samples, followed by centrifugal concentration at 42 °C. CE-TOFMS experiments were performed using an Agilent CE Capillary Electrophoresis System (Agilent Technologies, Santa Clara, CA, USA). Data acquisition was performed using Metabolome Analysis and Screening Tool for Easy and Rapid HANDling of Sample data (Master Hands) (ver. 2.17.2.15, Keio University, Tsuruoka, Yamagata, Japan).

### 4.10. ATP Assay

DLD-1 cells were seeded at a concentration of 0.5 × 10^5^ cells/ml in 6-well plates on the day before transfection. After 24 h, the cells were transfected with 5 nM control RNA, siR-*PTBP1*, siR-*hnRNPA1*, or siR-*SRSF3* for 72 h. ATP production was measured with an ATP Determination Kit according to the manufacturer’s protocol (Life Technologies). ATP production was normalized to cell numbers.

### 4.11. Lactate Assay

The same cells and siRNA treatment protocol as used for the ATP assay were used to assess lactate levels, which were measured with an l-Lactate Assay Kit according to the manufacturer’s protocol (Cayman Chemical Company, Ann Arbor, MI, USA). Lactate production was normalized to the number of cells.

### 4.12. Morphological Assessment of ROS Production

DLD-1 cells were stained with 5 µM MitoSOX™ Red mitochondrial superoxide indicator (Life Technologies) for 10 min at 37 °C, washed once with PBS, and examined by fluorescence microscopy using an Olympus fluorescence microscope (Tokyo, Japan) equipped with an epi-illuminator and appropriate filters.

### 4.13. Electron Microscopy Study

DLD-1 cells transfected with control RNA, siR-PTBP1, siR-hnRNPA1, or siR-SRSF3 at 72 h were rinsed with PBS. The cells were then fixed for 2 h with 2% paraformaldehyde and 2.5% glutaraldehyde in 0.2 M phosphate buffer (pH 7.4, PB), rinsed in PB, and postfixed in 2% osmium tetraoxide for 2 h. After having been washed with PB, the cells were progressively dehydrated by passage through a 10% graded series of 30%–100% ethanol and then cleared in QY-1 (Nissin EM, Tokyo, Japan). Thereafter, they were embedded in Epon 812 resin (TAAB Laboratories Equipment, Reading, UK) and thin sections (70 nm thickness) were prepared. Finally, the sections were stained with uranyl acetate and lead citrate and examined by transmission electron microscopy with a Hitachi-7650 (Hitachi, Tokyo, Japan), operating at 80 kV.

### 4.14. Human Tumor Xenograft Model

All animal experiments were approved by the Committee for Animal Research and Welfare of Gifu University (Approval number: 29-53, 3 August 2017). Female athymic nude mice (BALB/c SLC-nu/nu) were obtained from Japan SLC (Hamamatsu, Japan). Twelve nude mice were inoculated subcutaneously into each of their flanks, with DLD-1 cells at 1 × 10^6^ in 100 µM PBS. After 10 days, the inoculated-DLD-1 formed tumors (Day 0). The control group (*n* = 6) and siRNA-*SRSF3*-injected group (*n* = 6) received control RNA and siRNA-*SRSF3* (560 µg/kg per 1 administration) into their heart every 3 days for 5 times. For injection, siRNAs were mixed with poly (ethylene glycol)-poly (Ornithine), a gift from Professor Kataoka (Kawasaki Institution of Industrial Promotion Innovation Center of NanoMedicine), to obtain siRNA-loaded polyion complexes (PICs) to deliver siRNAs to the tumor. Tumor volume was monitored by measuring the long length (*L*_1_) and short length (*L*_2_), and estimating volumes according to the following formula: *V*(mm^3^) = 0.5236*L*_1_(*L*_2_)^2^.

### 4.15. RIP Assay

RIP assay was performed according to the manufacturer’s protocol, provided in a RiboCluster Profiler™ RIP-Assay Kit (MBL). DLD-1 cells were seeded at a concentration of 0.5 × 10^5^ cells/ml in 6-well plates on the day before transfection. After 24h, the cells were transfected with 5 nM control RNA, siR-*PTBP1*, siR-*hnRNPA1*, or siR-*SRSF3* for 72 h under an atmosphere of 95% air and 5% CO_2_ at 37 °C. Antibodies against the following antigens were used: PTBP1 and hnRNPA1 (Cell Signaling Technology) and SRSF3 (MBL). After RNA extraction, the RNA was reverse-transcribed with a PrimeScript RT Reagent Kit (Takara Bio Inc.). The cDNA used as template of PCR that was performed according to the manufacturer’s protocol provided in a Platinum™ SuperFi™ DNA polymerase (Life Technologies). The product of PCR was loaded onto 2% agarose gels (Takara Bio Inc.) and visualized by staining with ethidium bromide. The primers for PKM gene set 1, 2, 3. and 4 were the following: Set 1-Forward, 5′-AATACGACTCACTATAGGGATAGCTCGTGAGGCTGAGGC-3′; and set 1-Reverse, 5′-GCTCGATCGAGGCGTGCCTGCCAGACTCCGTCAGAA-3′; set 2-Forward, 5′-AATACGACTCACTATAGGGATTGCCCGTGAGGCAGAGGC-3′; and set 2-Reverse, 5′-GCTCGATCGAGGCGTGCCTGCCAGACTTGGTGAGGA-3′; set 3-Forward was the same as set 1 forward one and set 3-Reverse, 5′-GCTCGATCGAGGCGTGCTAGGGGAGCAACATCCGTC-3′; set 4-Forward, 5′-AATACGACTCACTATAGGGTAGGGCCCTAAGGGCAGGTA-3′; and set 4-Reverse was the same as set 2 reverse one. The bands densitometry was calculated by using ImageJ.

### 4.16. Nuclear Protein Extraction and Crosslink-Immunoprecipitation

Protein G agarose beads (Life Technologies) were incubated with 5 µg of anti-PTBP1 or anti-SRSF3 IgG in 20 mM HEPES for 1 h at 4 °C. After centrifugation at 2500× *g* for 1 min, the supernatant was discarded and then 110 µg of the cross-linking reagent DSS (Life Technologies) in 20 mM HEPES (Sigma-Aldrich) was added. After 30 min at room temperature, the crosslinking reaction was quenched by adding 33 mM Tris-HCl (Tris; Life Technologies, HCl; Wako) and incubating for 15 min at room temperature. Nuclear protein extraction was performed according to the manufacturer’s protocol, provided in a CelLytic™ NuCLEAR™ Extraction Kit (Sigma Aldrich). Lysates were incubated with the antibody-conjugated protein G agarose beads overnight at 4 °C. The beads were then washed 3 times with each washing buffer (5 × Lysis Buffer isotonic with 1% NP-40 (Sigma-Aldrich) or 20 mM HEPES, pH 7.9, containing 1.5 mM MgCl_2_ (Wako), 0.42 M NaCl (Wako), 0.2 mM EDTA (Life Technologies), 25%(*v*/*v*) glycerol (Sigma-Aldrich) with 1% (NP-40). The bound proteins were eluted with 2 × sample buffer without DTT for 10 min at 50 °C. After centrifugation at 2500× *g* for 1 min, we transferred the supernatant and added DTT (Wako) at 100 mM into the supernatant (Elution 1). The pellet beads were added 2 × sample buffer with 100 mM DTT for 5 min at 98 °C. After centrifugation at 2500× *g* for 1 min, we then transferred the supernatant into a new tube (Elution 2).

### 4.17. Statistics

Each examination was performed in triplicate. Differences were statistically evaluated by the *t*-test. Data were presented as means +/± SD. A *p* value of less than 0.05 was considered to be statistically significant.

## Figures and Tables

**Table 1:** Clinicopathological features in colorectal cancer patients.

Case	Age	Sex ^a^	Site ^b^	Size ^c^	Depth ^d^	Stage ^e^
1	41	M	RS	70×45	SS	B
2	49	F	R	50×70	SS	B
3	42	M	R	30×20	SS	B
4	68	M	R	65×60	SE	D
5	73	M	R	40×40	MP	A
6	56	F	S	27×24	SS	C
7	64	M	R	28×22	MP	A
8	82	F	A	33×28	MP	A
9	62	M	S	55×40	SS	D
10	68	F	R	35×35	SS	B

^a^ M, male; F, female. ^b^ Location of tumor: RS, rectosigmoid; R, rectum; S, sigmoid colon; A, ascending colon. ^c^ Diameter in mm. ^d^ SS, Subserosa; SE, Serosa exposure; MP, Mucosa propria. ^e^ Duckes’ system classification.

**Table 2:** Clinicopathological features in colorectal adenoma patients.

Case	Sex ^a^	Site ^b^	Size ^c^	Grade ^d^
1	F	S	20	High
2	F	S	20	Low
3	F	A	15	Low
4	M	S	15	Low
5	M	R	10	High

^a^ M, male; F, female. ^b^ Location of tumor: S, sigmoid colon; A, ascending colon; R, rectum. ^c^ Diameter in mm. ^d^ Low, low-grade adenoma (mild and moderate atypia); High, high-grade adenoma (severe atypia and carcinoma in situ).

## Supplementary Materials

Supplementary materials can be found at http://www.mdpi.com/1422-0067/19/10/3012/s1.

## Abbreviations

SRSF3	serine and arginine rich splicing factor 3
SR	serine and arginine
PTBP1	polypyrimidine tract binding protein 1
hnRNP	heterogeneous nuclear ribonucleoprotein
PKM	pyruvate kinase muscle
TCA	tricarboxylic acid
ADP	adenosine 5′-diphosphate
ATP	adenosine 5′-triphosphate
PEP	phosphoenolpyruvate
AS	alternative splicing
ESE	exonic splicing enhancer
OXPHOS	oxidative phosphorylation
GAPDH	glyceraldehyde-3-phosphate dehydrogenase
ROS	reactive oxygen species
ESS	exonic splicing silencer
ISS	intronic splicing silencer
ISE	intronic splicing enhancer
FOXM1	forkhead box protein M1
