# Supplementary material for: SRSF3, a Splicer of the PKM Gene, Regulates Cell Growth and Maintenance of Cancer-Specific Energy Metabolism in Colon Cancer Cells
Source: Int J Mol Sci. 2018 Oct 2;19(10):3012. doi: 10.3390/ijms19103012 (PMC6213643; doi:10.3390/ijms19103012)
Supplement: Supplementary file 1 [file ijms-19-03012-s001.pdf]

SRSF3, a splicer of the *PKM* gene, regulates to cell growth and maintenance of cancer-specific energy metabolism in colon cancer cells

Yuki Kuranaga [1], Nobuhiko Sugito [1], Haruka Shinohara [1], Takuya Tsujino [2], Kohei Taniguchi [3,4], Kazumasa Komura [2,4], Yuko Ito [5], Tomoyoshi Soga [6] and Yukihiro Akao [1,*]

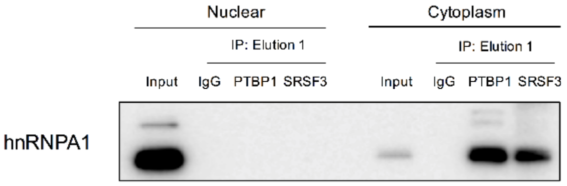

Supplementary Figure 1. Western blot of hnRNPA1 from the immunoprecipitated samples. The detail is shown in Materials and Methods section.
